# Transcranial Sonography in the Diagnosis of Pituitary Tumor—A Direct Comparison With MRI

**DOI:** 10.3389/fendo.2021.778839

**Published:** 2021-12-03

**Authors:** Lei He, Jinghan Zhang, Tengfei Yu, Yue Du, Xinyao Liu, Wen He

**Affiliations:** ^1^ Department of Ultrasound, Beijing Tiantan Hospital, Capital Medical University, Beijing, China; ^2^ Beijing Tiantan Hospital, Capital Medical University, Beijing, China

**Keywords:** ultrasonography, Doppler, transcranial, MRI, Sella turcica, pituitary neoplasms, comparative study

## Abstract

**Background:**

Transcranial sonography (TCS) is a convenient tool for detecting certain brain diseases, such as brain tumors. Few studies have reported on the use of TCS in the area of Sella turcica. The accuracy and repeatability of Sella turcica with or without pituitary tumor is not clear.

**Purpose:**

This study aimed to investigate the feasibility and accuracy of TCS to measure the size of Sella turcica according to the measurement in MRI and determine its diagnostic performance in individuals with pituitary tumor.

**Materials and Methods:**

In this cross-sectional comparative study, healthy volunteers and patients with pituitary tumor were enrolled for examination of TCS and MRI between October 2020 and July 2021. The transverse diameter (D1, cm) of Sella turcica and the volume of the pituitary tumor were measured by TCS and MRI, respectively, and compared by using Student’s *t*-test or Mann–Whitney test, using the receiver operating characteristic (ROC) curve to analyze the diagnostic value of D1 in TCS for pituitary tumor.

**Results:**

A total of 75 healthy volunteers and 51 patients with pituitary tumor were evaluated. In healthy volunteers, the mean D1 was 1.30 ± 0.35 (range, 0.82–3.22) by TCS and 1.32 ± 0.29 (range, 0.94–3.02) by MRI (*P* = 0.054). In patients with pituitary tumor, the mean D1 was 2.0 ± 0.65 (range, 0.90–3.48) by TCS and 2.42 ± 1.0 (range, 0.80–4.70) by MRI (*P* = 0.000). The median measurement volume was 4.41 and 6.59 cm^3^ in TCS and MR, respectively (*P* = 0.000). The mean D1 was 1.31 ± 0.35 in healthy volunteers and 2.0 ± 0.65 cm in patients with pituitary tumor (*P* = 0.000). In the ROC curve analysis, the area under the curve was 0.836, and the optimal cutoff value (1.56) exhibited a sensitivity and specificity of 67.31 and 88.0%, respectively.

**Conclusion:**

The consistency between the two imaging technologies performed well in D1 measurement, while the volume of the pituitary tumor was smaller as assessed by TCS than by MRI. D1 in TCS had good diagnostic performance in pituitary tumor.

## Introduction

The Sella turcica is a saddle-shaped structure in which the anterior and posterior walls are formed by a part of the sphenoid bone. The cavernous sinus forms its lateral wall, which is traversed by the internal carotid artery. We found that transcranial sonography (TCS) can detect the Sella turcica structure which appeared as a hyperechoic ring. However, little literature has reported these features.

The pituitary gland is surrounded by the Sella turcica. In normal populations, the pituitary volume has been found to increase with age (1). After 26 years old, the size is usually stable with no further significant increase. According to other literature (2), the dimension of the Sella turcica is approximately that of the pituitary gland among healthy people. An abnormal pituitary gland is usually manifested as changing in size, resulting in Sella turcica enlargement, such as an empty Sella turcica (3, 4).

An enlarged pituitary gland is associated with pituitary tumors, traumatic brain injury, and disorder of functions (5). Pituitary tumor is the most common problem with an overall prevalence rate of 16.7% (6). Although MRI is the most widely used method to diagnose pituitary tumors, this technique is quite costly and time-consuming, especially since the pituitary gland requires specialized imaging sequences. In addition, MRI has contraindications for electronic or metal implants (such as pacemakers, cochlear implants, aneurysm clips, and surgical devices) (7) and claustrophobia.

Transcranial sonography is a fast, portable, repeatable, and cost-effective examination with which it is possible to observe the morphological appearance of the Sella turcica and the pituitary tumor directly by the transtemporal bone window, which is not mentioned in other literature as far as we know. The purpose of this study was to investigate the accuracy of the measurement and diagnostic performance of TCS on Sella turcica and pituitary tumors.

## Materials and Methods

### Study Population

Patients with pituitary tumors and healthy volunteers were recruited in our study from Beijing Tiantan Hospital between October 2020 and July 2021. The age of all subjects was greater than 26 years old, as the size of the Sella turcica is stable after this age (8, 9). Patients with or without pituitary tumors were diagnosed by MRI. Then, all patients underwent TCS examination, and the time period between the two examinations did not exceed 1 week. All patients with pituitary tumors had undergone surgery and were histologically confirmed.

This study was approved by the Research Ethics Board of Beijing Tiantan Hospital, Capital Medical University, and the requirement for written informed consent was waived.

### Measurement Definition

**Figure 1:**
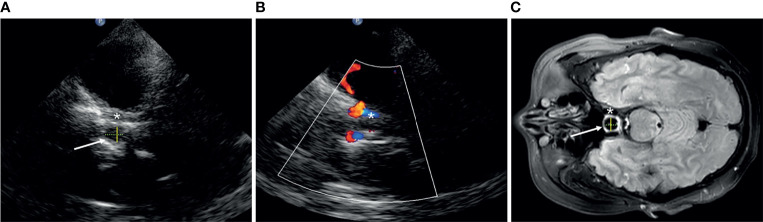
The structure of Sella turcica (arrow) in transcranial sonography **(A, B)** and MRI **(C)**. Asterisk, cavernous segment of the internal carotid artery; yellow solid line, D1; green dotted line, D2.

We define the transverse diameter (D1, cm) and the anterior–posterior diameter (D2, cm) of the Sella turcica as the inner side of two hyperechoic lines between the cavernous internal carotid artery and the bony anterior and posterior wall, respectively, in the axial position (**Figure 1**). The vertical diameter was not definite because it cannot be identified by TCS. The pituitary tumor volume (PV, cm^3^) = anteroposterior diameter × superior–inferior diameter × left–right diameter × 0.52, both in TCS and MRI.

### Data Collection

After enrollment, the sex and the age of each patient were recorded. D1 and D2 in all patients were measured both in MRI (T2-weighted MR images) and TCS, respectively, by two experienced radiologists who were blinded to the clinical and MR information of the patients when performing the TCS examination. Three diameters were measured in patients with pituitary tumor, and then the volume was calculated according to the formula given above. The TCS characteristics of the pituitary tumors were described through echogenicity and the integrity of the Sella bone.

### TCS and MRI Examination Technique

All patients underwent transcranial ultrasound examination by a phased-array ultrasound system equipped with a 1.6–2.5 MHz transducer (EPIQ 7, Philips Medical Systems). A dynamic range of 45–55 dB and a penetration depth of 14–16 cm through the transtemporal bone window were used (10). Image brightness and time gain compensations were adapted as needed for each patient.

Firstly, with the patient lying in a supine position, the plane of the mesencephalic brainstem is insonated through the transtemporal bone window parallel to the imagined orbito-meatal line. By tilting the probe slightly downward for about 10-20°, the Sella turcica plane is reached. For assessing the pituitary tumor, the probe was tilted upward slowly to find the maximum plane of the tumor and measure the anteroposterior and left–right diameter, and then the probe was rotated 90°clockwise to measure the superior–inferior diameter.

The T2-weighted images of all individuals were obtained with a Trio 3.0T scanner (Siemens). The T2-weighted image parameters were as follows: TR = 5,800 ms; TE = 110 ms; flip angle = 150°; field of view = 240 × 188 mm^2^; voxel size = 0.6 × 0.6 × 5.0 mm^3^; matrix = 384 × 300. All scans were acquired without an intravenous contrast agent and with the patient in a supine position during end inspiration.

### Statistical Analysis

We recorded the measured values ​​of the two radiologists separately and then took the average of their measurements as the final statistical data. To evaluate intra- and interoperator reproducibility, the interclass correlation coefficient (ICC) was calculated using both TCS and MRI measurements of D1 and PV. Measurement reliability was classified according to common criteria as excellent (ICC > 0.75), good (ICC = 0.60–0.75), fair (ICC = 0.40–0.59), and poor (ICC ≤ 0.40) (11). Qualitative variables were expressed by number (percentage) and tested using the chi-square test. Qualitative variables were expressed by number (percentage) and tested using the chi-square test. For quantitative variables, the normality of distribution was tested using Shapiro–Wilk test and expressed as mean (SD) or median (interquartile range). Unpaired quantitative data were compared using Student’s *t*-test or Mann–Whitney test. Paired quantitative data were compared using paired Student’s *t*-test or Wilcoxon test.

Calculated *P*-values less than or equal to 0.05 were considered statistically significant for all two-sided tests.

SPSS (IBM^®^ SPSS^®^ Statistics Version 26) statistical software was used for all data analyses. Receiver operating characteristic (ROC) curve analysis for predicting pituitary tumor by D1 was performed using MedCalc software (MedCalc^®^ Version 19.6.1).

## Results

**Figure 2:**
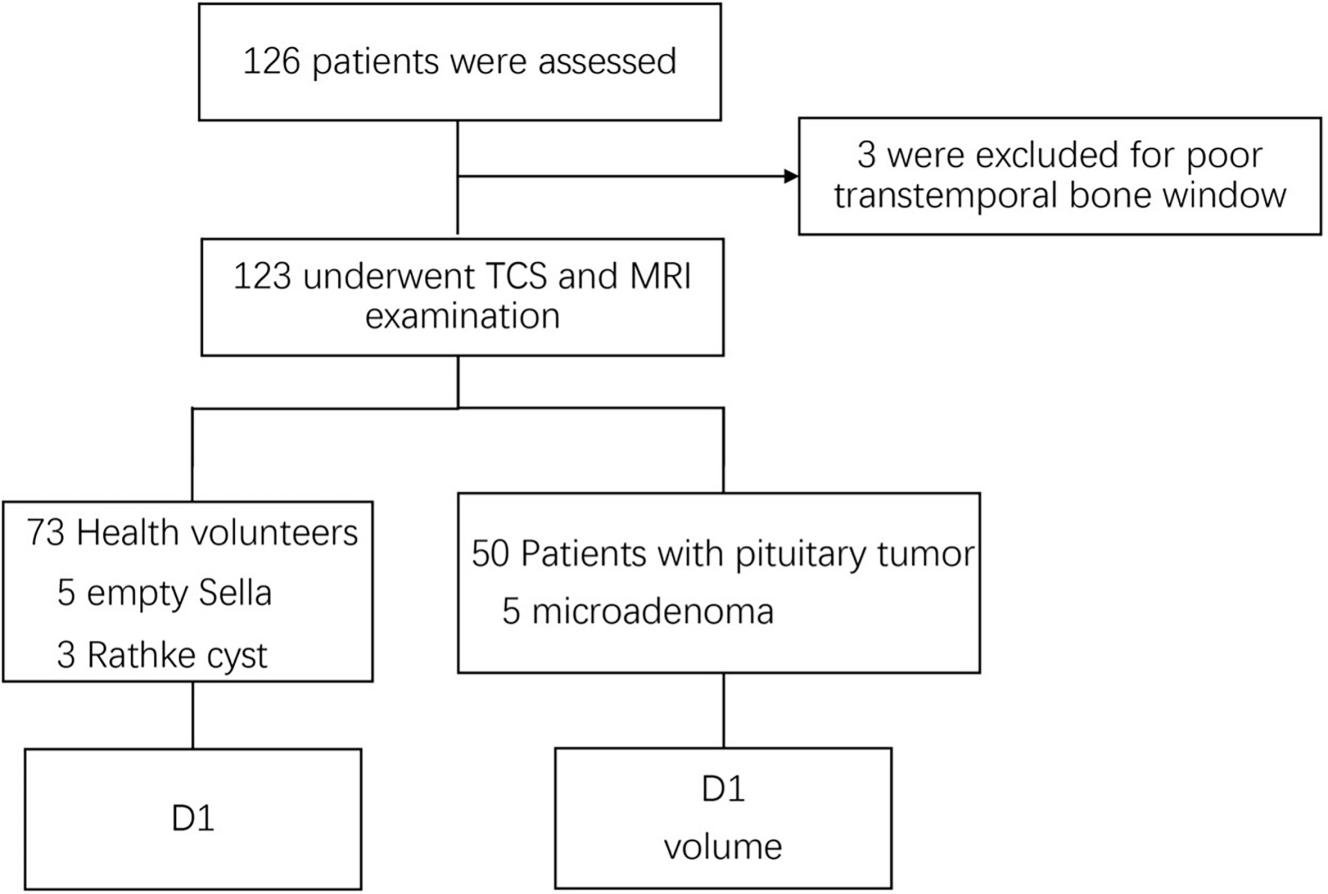
Flow diagram of the inclusion of patients in the study. D1, transverse diameter.

A total of 126 patients aged from 28 to 76, including 51 patients with pituitary tumor and 75 healthy volunteers, were recruited. Three patients (two volunteers and one patient) were excluded because of a poor transtemporal bone window (**Figure 2**). Therefore, a total of 123 patients were analyzed: 50 were patients with pituitary tumor and 73 were healthy volunteers (**Tables 1**, **2**). The intra- and interoperator ICCs for the measurements of D1 and PV using TCS and MRI are 0.911 and 0.902 and 0.954 and 0.931, respectively.

**Table 1:** Demographics and characteristics of transcranial sonography patients.

	Healthy volunteer (*n* = 73)	Patients with pituitary tumor (*n* = 50)	*P*-value
Age (years)^a^	51 ± 12	53 ± 12	0.31
Sex (woman)	32 (42.7%)	22 (41.5%)	0.89
D1 (cm)^a^	1.30 ± 0.35	2.00 ± 0.65	<0.01
Echo (h/e/l)		24 (48%)/17 (34%)	

a Data are means ± standard deviation. h/e/l, high echo/equal echo/low echo.

**Table 2:** Comparison of measurements between transcranial sonography (TCS) and MRI.

	TCS	MRI	*P*-value
Healthy volunteer			
D1^a^	1.30 ± 0.35	1.32 ± 0.29	0.05
Patients with pituitary tumor			
D1^a^	2.00 ± 0.65	2.42 ± 1.0	<0.01
Volume (cm^3^)^b^	4.41 (1.85–8.47)	6.59 (2.23–12.83)	<0.01

a Data are means ± standard deviation.

b Data are medians, with interquartile ranges in parentheses.

### Healthy Volunteer

There were 32 women (42.7%) and 43 men (57.3%) in this group. The mean age was 51 ± 12 years. The anterior–posterior hyperechoic lines cannot be visualized by TCS in most cases, both in patients with pituitary tumor and in healthy volunteers, so we only compared D1 between the two groups. The average distance of D1 was 1.30 ± 0.35 (range, 0.82–3.22) by TCS and 1.32 ± 0.29 (range, 0.94–3.02) by MRI (*P* = 0.053), respectively, which subsume five empty Sella and three Rathke cyst. The median distance of D1 was 1.36 in women and 1.20 in men (*P* = 0.051).

### Patients With Pituitary Tumor

**Figure 3:**
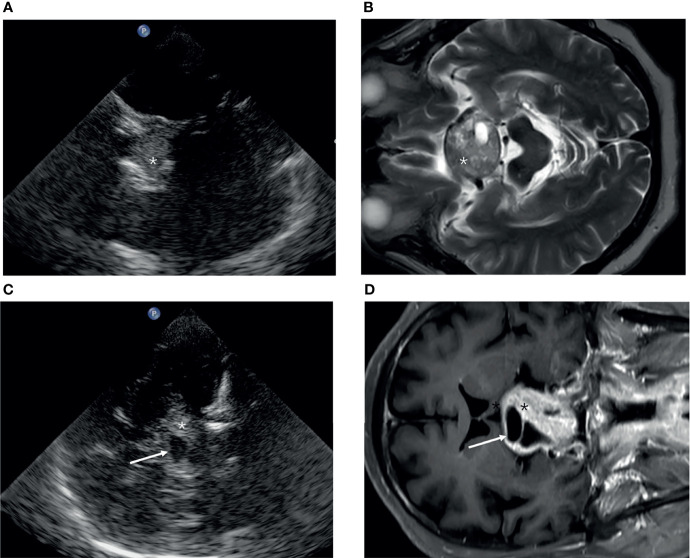
The appearance of a pituitary tumor (asterisk) in transcranial sonography **(A, C)** and MRI **(B**, **D)**. **(A, B)** Axial plane. **(C, D)** Coronal plane.

The cohort consisted of 22 women (41.5%) and 28 men (58.5%), with a mean age of 53 ± 12 years. Five tumors were not visualized because of pituitary microadenoma, and the remaining 45 were detected (90%). The pituitary tumors (**Figure 3**) were hyperechoic in 24 cases (48%), hypoechoic in nine cases (18%), and isoechoic in 17 cases (34%). Bone destruction of the Sella turcica was observed in five cases. In nine cases, the tumor extruded the cavernous ICC and caused ICC displacement. The average distance of D1 was 2.0 ± 0.65 (range, 0.90–3.48) by TCS and 2.42 ± 1.0 (range, 0.80–4.70) by MRI respectively (*P* = 0.000). The median measurement volume was 4.41 and 6.59 cm^3^ in TCS and MR, respectively (*P* = 0.000).

### Comparison of D1 Between Two Groups

The average distance of D1 was 1.31 ± 0.35 in healthy volunteers and 2.0 ± 0.65 in patients with pituitary tumor (*P* = 0.000).

### D1 Prediction for Pituitary Tumor

**Figure 4:**
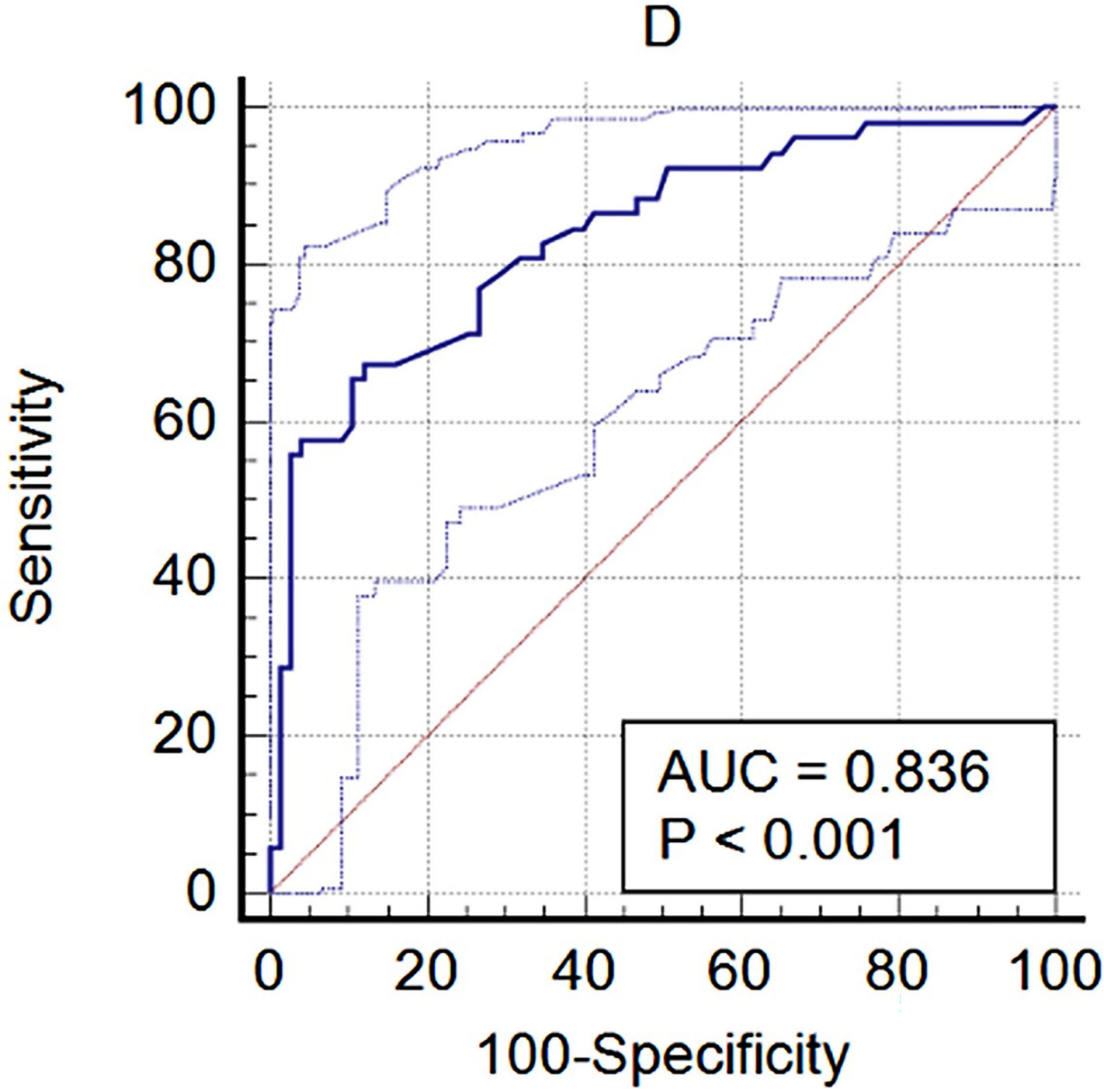
The diagnostic performance of D1 with pituitary tumor.

D1 was used to predict pituitary tumor, and a ROC curve was delineated (**Figure 4**). In the ROC curve analysis, the area under the curve was 0.836, and the optimal cutoff value (1.56) exhibited a sensitivity and specificity of 67.31 and 88.0%, respectively.

## Discussion

To the best of our knowledge, this is the first paper to describe the characteristics of Sella turcica and pituitary tumor in TCS and analyze its diagnostic performance. MRI is the preferred imaging method for detecting pituitary tumors, though sometimes PET is also used in cases when it is hard to diagnose with MRI (12), so we tried to compare these two imaging techniques in order to assess the performance of TCS in detecting Sella turcica abnormality.

We found that the Sella turcica typically appears as a hyperechoic ring on TCS. However, in most cases, only the two hyperechoic lines of the lateral walls were detected. Anatomically, the anterior and posterior walls compose the sphenoid bone which is hard, and the lateral wall of the cavernous sinus is relatively soft, so we supposed that an enlarged pituitary gland could affect the lateral walls of the Sella turcica more easily. In addition, we cannot observe the height of the Sella turcica or the pituitary gland, and Ranganathan (3) found that the heights of the gland and of the Sella were not significantly statistically different in the controls and idiopathic intracranial hypertension patients. Therefore, we choose the dimension of the lateral wall (D1) as the size of the Sella turcica.

The detection rate of the Sella turcica by TCS in this study is 97.6%, which is amazingly high. Even with a quite poor temporal bone window, TCS could also make the Sella turcica appear. D1 showed no significant differences between the two measurements by TCS and MRI in healthy volunteers, whereas the volume of the pituitary tumor in TCS was smaller than in MRI. It is confusing because the tumor borders were mostly clear. The reason may be sound attenuation which was too severe when the sound waves penetrate the skull, resulting in insufficient resolution.

The ROC curve showed that D1 had a high diagnostic value on the pituitary tumor. The specificity was 88.0%; however, the sensitivity was only 67.31%, which means a higher false positive. A pituitary tumor is not the only reason for an enlarged Sella turcica. Obesity (13) and even growth hormone deficiency (14) can also induce an enlarged Sella turcica. In our study, there were five empty Sella and three Rathke cysts in healthy volunteers. These cases reduced the sensitivity of D1 for diagnosing pituitary tumor. The optimal cutoff value was 1.56 cm, which was reasonable because the normal Sella turcica ranges from 0.4 to 1.2 cm in previous radiographic studies (9).

Our study had several limitations. First, it was a cross-section study performed in a single institution. The number of subjects was not large enough. Second, selection bias may have existed when choosing the patients with pituitary tumor. The healthy volunteers comprise empty Sella and Rathke cyst. Third, although this was a blinded study, the examiners may be influenced subjectively on measuring D1 as a pituitary tumor was easy to detect by TCS.

Although TCS allows a quick, reliable, and inexpensive depiction of the Sella turcica and pituitary tumor, it is an operator-dependent examination. A learning curve is mandatory not only to perform the exam but also to understand the images. In our experience, TCS can be learned within 1 week and be reliably performed after completing the examination of 50 patients under the supervision of an expert. TCS is more suitable for screening an abnormality of the Sella turcica and cannot be regarded as a substitute for MRI. Sella turcica abnormality can be caused by several reasons, such as obesity, increased cranial pressure, or hormone abnormalities, as mentioned above. Further studies need to be done to extend the application of TCS in the field of Sella turcica, such as the recurrence of a pituitary tumor.

In conclusion, we first accessed the diagnostic performance of TCS to identity the abnormal Sella turcica caused by a pituitary tumor. Most pituitary tumors were hyperechoic. D1 was a good predictive factor for diagnosing pituitary tumors in TCS.

## Data Availability Statement

The original contributions presented in the study are included in the article. Further inquiries can be directed to the corresponding author.

## Ethics Statement

The studies involving human participants were reviewed and approved by the Research Ethics Board of Beijing Tiantan Hospital, Capital Medical University. Written informed consent for participation was not required for this study in accordance with the national legislation and the institutional requirements.

## Author Contributions

LH wrote the manuscript. JZ, TY, YD, and XL performed the experiments included in the manuscript. All authors contributed to the article and approved the submitted version.

## Funding

This work was supported by the National Natural Science Foundation of China (ID 81730050).

## Conflict of Interest

The authors declare that the research was conducted in the absence of any commercial or financial relationships that could be construed as a potential conflict of interest.

## Publisher’s Note

All claims expressed in this article are solely those of the authors and do not necessarily represent those of their affiliated organizations, or those of the publisher, the editors and the reviewers. Any product that may be evaluated in this article, or claim that may be made by its manufacturer, is not guaranteed or endorsed by the publisher.

## Abbreviations

TCS, transcranial sonography; D1, transverse diameter; D2, anterior–posterior diameter; ICA, internal carotid artery; PV, pituitary tumor volume; ROC, receiver operating characteristic; AUC, area under the curve.
